# The Application of Chemical Foaming Method in the Fabrication of Micro Glass Hemisphere Resonator

**DOI:** 10.3390/mi9020042

**Published:** 2018-01-24

**Authors:** Jianbing Xie, Lei Chen, Hui Xie, Jinqiu Zhou, Guangcheng Liu

**Affiliations:** Key Laboratory of Micro/Nano Systems for Aerospace, Ministry of Education, Northwestern Polytechnical University, Xi’an 710072, China; cl@mail.nwpu.edu.cn (L.C.); hhx@mail.nwpu.edu.cn (H.X.); zjq523543302@mail.nwpu.edu.cn (J.Z.); lgc1995@mail.nwpu.edu.cn (G.L.)

**Keywords:** hemisphere resonator gyroscope, hemisphere resonator, chemical foaming process, glassblowing, hemisphere shell, hollow glass microsphere, micro electro mechanical systems (MEMS)

## Abstract

Many researchers have studied the miniaturization of the hemisphere resonator gyroscope for decades. The hemisphere resonator (HSR), as the core component, has a size that has been reduced to the submillimeter level. We developed a method of batch production of micro-hemisphere shell resonators based on a glass-blowing process to obtain larger hemisphere shells with a higher ratio of height to diameter (H/D), we introduced the chemical foaming process (CFP) and acquired an optimized hemisphere shell; the contrasted and improved H/D of the hemisphere shell are 0.61 and 0.80, respectively. Finally, we increased the volume of glass shell resonator by 51.48 times while decreasing the four-node wineglass resonant frequencies from 7.24 MHz to 0.98 MHz. The larger HSR with greater surface area is helpful for setting larger surrounding drive and sense capacitive electrodes, thereby enhancing the sensitivity of HSR to the rotation. This CFP method not only provides more convenience to control the shape of a hemisphere shell but also reduces non-negligible cost in the fabrication process. In addition, this method may inspire some other research fields, e.g., microfluidics, chemical analysis, and wafer level package (WLP).

## 1. Introduction

The hemisphere resonator gyroscope (HRG), as one type of Coriolis gyro, has superior navigation precision because of its unique axisymmetric 3D structure [1]. Using a MEMS-based process, many different mHRGs and uHRGs have already been studied by global research teams. The different structural designs of the resonator mainly include hollow hemisphere [2], wineglass [3], disk [4], these resonators are usually surrounded by several coupled drive and sense electrodes. The fabrication processes of the resonator also differ, including deep reactive ion etching (DRIE), glassblowing [5], atomic layer deposition [6], sacrificial layer etching [7], or a combination of the above methods.

Quality factor (Q-factor) is a dimensionless characteristic, which is defined as the ratio of the energy stored to the power loss in every cycle when a system works on resonant status. The hemisphere resonator (HSR) with a high Q is desired because it can reduce mechanical noise. The Q factor of the HSR mainly depends on thermoelastic damping and anchor losses, studies of the detailed energy loss mechanisms were presented in [7,8]. A low thermoelastic damping structural material can achieve a high Q factor; for example, an HSR fabricated by microcrystalline diamond was found to have a four-node wineglass resonant frequency of 18.321 kHz, with the observed Q of ~10,000 at the four-node wineglass mode and 20,000 at six-node vibration mode [9]. A stem-supported hemispherical shell with self-aligned tall capacitive electrodes was fabricated in polysilicon; the shell has a wineglass resonant mode at 5.58 kHz with a Q of 17,600 [10]. Other materials, such as fused silica [11] and Pyrex have also been used to fabricate an HSR [2]. The ideal drive axis and sense axis of a gyroscope should be mutually perpendicular, which is called quadrature coupling, but the fabrication imperfections, such as lithographic misalignment and inhomogeneous anchor region, would prevent the angle of drive axis and sense axis from being 90° and result in quadrature error. When compared with the conventional 2D flat massive block typed gyroscope, the inherent central symmetry resonator can reduce quadrature error and provide more flexible signal excitation and pick up design.

**Figure 1:**
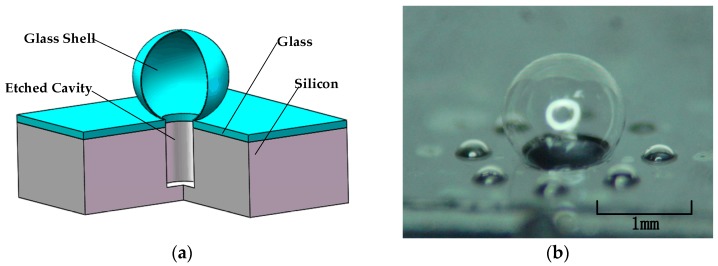
Demonstration diagram and micrograph of the glass hemisphere resonator (HSR): (**a**) cross-sectional view of the micro glass shell structure; (**b**) microphotograph of a glass-blown shell (height/diameter (H/D) = 0.79).

A micro-hemisphere glass shell resonator is a good choice as the central component of the HRG. Figure 1a shows a typical structure demonstration of a glass HSR, which has many advantages, such as high stiffness, good shock resistance, and low thermal expansion coefficient consistent with that of a silicon substrate [12]. The silicon substrate also provides high aspect ratio surrounding electrodes and enables simultaneous fabrication [13]. Moreover, the processes for batch fabrication this type HSR have been developed in [5], the key processes (which include DRIE, anodic bonding, and glass blowing) are simple and convenient techniques those are easily accessible to related research groups. For example, a 500-μm radius spherical resonator with a four-node wineglass resonant frequency of 1.37 MHz and a Q factor of 1280 in vacuum (0.4 mT) was reported in [6]. Further Q enhancement is possible by minimizing the shell anchoring to the substrate or by using high-Q materials [2].

At the beginning, we utilized a similar process to fabricate the micro hemispherical glass shell, however, the shell shapes were not very uniform. Figure 1b shows the highest hemisphere glass shell blown by an 800 μm deep cavity, the radius of the etched cavity is 250 μm. We measured the shape of the glass HSR via scanning electron microscope (SEM), the radius of this HSR is 439.18 μm. We calculated that the glass HSR has a ratio of height to diameter (H/D) of 0.79. However, it is difficult to obtain such a large glass shell because the H/D of most other samples did not exceed 0.5. An HSR with high H/D is desirable for the larger HSR has several advantages, for example, greater surface area is helpful for setting higher aspect surrounding drive and sense capacitive electrodes, which can increase the sensitivity of HRG, and HSR with high H/D would lower its four-node wineglass resonant frequency, which is beneficial for the drive and sense of the HRG. Finally, we managed to develop an available method to produce larger, uniform glass HSRs.

There are some challenges in the fabrication of a glass HSR, for example, very deep cylindrical cavities (usually 800 μm in depth) must be etched previously, requiring a thick silicon substrate (equal or greater than 1000 μm in thickness). This thickness requirement not only increases the process time and cost but also influences the subsequent processes because the excessively thick silicon substrate increases the difficulty of the anodic bonding and dicing processes. Even with the use of such a deep cavity to blow a hemispheric glass shell, the volume of the glass shell is still under restrictions from the process parameters; thus, we seek a larger hemisphere glass shell by improving the process condition or introducing some other valid approaches. To get a micro glass hemisphere shell with higher H/D, Andrei M. Shkel et al. presented an alternative fabrication process to make an extra trapped air pocket [2,5], utilizing two bonded silicon wafers to achieve larger sphericity (H/D), because which can provide more volume of the sealed gas. D. Senkal et al. also utilized an additional stencil silicon layer to create an air pocket and fabricated a spherical shell structure with a stem, which was conventional micro machining technology [14]. Jintang Shang et al. used foaming agent TiH_2_ to fabricate glass bubbles [15] and hemispherical glass shells with self-formed stems [16]. To obtain a larger glass HSR, we managed to introduce chemical foaming process (CFP) method in the fabrication of glass-blown shell resonators, the H/D of glass hemisphere shell blown by 200 μm deep etched cavity with quantified foaming agent can approach (even exceed) the HSR blown by the 800 μm deep etched cavity without foaming agent.

**Figure 2:**
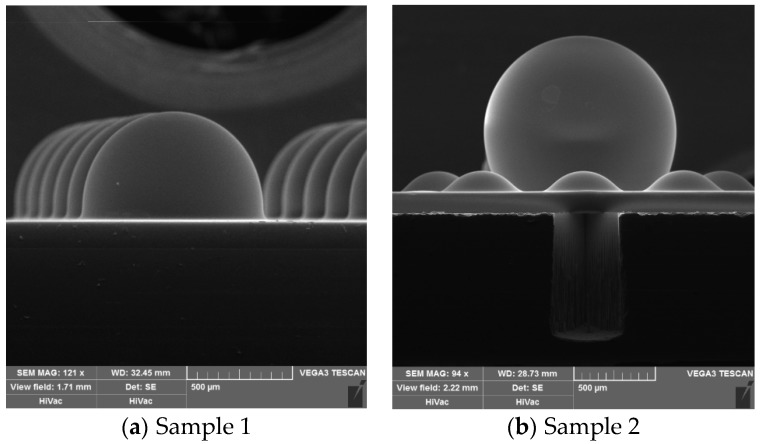
Scanning electron microscope (SEM) images of two different glass shells: (**a**) glass shell blown via an etched cavity without a foaming agent (H/D = 0.61); (**b**) glass shell blown via an etched cavity with quantified foaming agent (H/D = 0.80).

In this work, analytical model and experimental verification were presented sequentially. We utilized the height model to predict the shape of the glass-blown spherical shell, and then correlative bonded stacks were heated in the furnace at a temperature above the softening point of the glass. In the initial experiment stage, the resulting shapes were almost hemispheric shells (Figure 2a), and the H/D of the shell samples was 0.61. We want to acquire a hollow glass shell that is closer to a sphere, because this structure could provide more desirable properties of the gyroscope. Finally, we explored an available method of transferring quantified foaming agent into the etched cavity before anodic bonding to obtain a larger shell very close to a sphere (Figure 2b). The two samples’ detailed experimental parameters are shown in Table 1 and characters are shown in Table 2.

## 2. Height Model of a Micro Glass-Blown Shell

**Figure 3:**
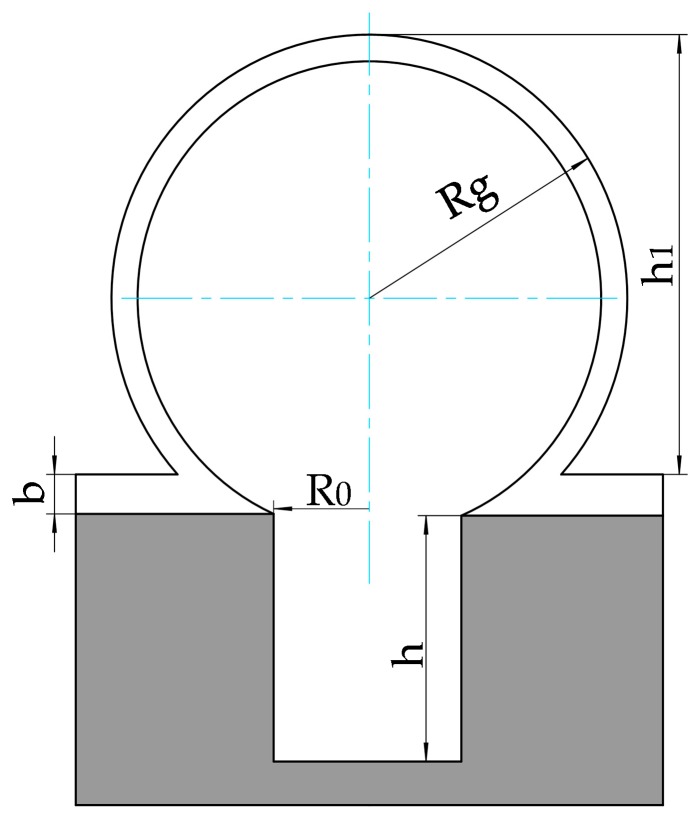
Height model of a micro glass-blown shell.

We have a height model to estimate the height of a glass-blown shell produced by an etched cavity with quantified foaming agent (Figure 3). The volume and shape of glass shell structures have been studied in [5], however, that study did not involve foaming agent. At a high temperature above the softening point of glass, the gas in the sealed cavity also includes the gas produced by foaming agent previously added in the etched cavity, this gas expands and increases the inner gas pressure before driving the high-temperature molten glass membrane to deform into a hollow spherical shell via the same surface pressure distribution. After 2–5 min the glass shell samples were removed rapidly to cool down in air to avoid the collapse of the glass shells. The shell shape was determined by the radius and depth of the etched cavity, the thickness of the glass wafer, the temperature at which the cavity was sealed, the temperature at which the glassblowing was executed, the mass of the foaming agent in sealed cavity, and even the cooling down process of the softened glass shell all have a significant influence on the final shape of the glass shell.

In the height model, we ignore the influence of gravity and the viscous force of the softened glass and assume the thickness of the glass shell is uniform; thus, the volume of inflated gas enclosed obeys the ideal gas law

*PV* = *nRT* (1)

where *P* is the inner pressure in the glass shell that is equal to atmosphere when the glass shell heated in furnace reaches balanced state; *n* is the number of moles, consisting of two components, one is the gas inside the etched cavity when bonding a glass wafer and the other is the gas released by the foaming agent; *R* is the Boltzmann constant; *T* is the temperature in furnace. Because the foaming agent provides extra molding gas, the equation can transform into

(2) $$V_{g}=\left(\frac{T_{f}}{T_{b}}-1\right)V_{E}+V_{F}$$

where *V_g_* is the volume of the glass shell, *T_f_* is the heating temperature in the furnace, and *T_b_* is the temperature at which the etched cavity was bonded to a glass wafer. *V_E_* is the volume of the etched cylindrical cavity, which can be written as

(3) $$V_{E}=\pi R_{0}^{2}h$$

where *h* is the depth of etched cavity. Regarding *V_F_*, which is the volume of gas released by foaming agent when the bonded wafer transferred in furnace set a high temperature of *T_f_*, we use CaCO_3_ as the blowing agent, the chemical decomposition of which is described as

(4) $${\mathrm{CaCO}}_{3}\left(s\right)\overset{\sim 900\;{}^{\circ}C}{=}\mathrm{CaO}\left(s\right)+{\mathrm{CO}}_{2}\left(g\right)$$

The means to transfer the foaming agent will be explained in later section. We can control *V_F_* by changing the number of moles of CaCO_3_, *n*_0_, which is equal to the number of moles of CO_2_; thus, *V_F_* can be derived as

(5) $$V_{F}=\frac{n_{0}RT_{f}}{P}$$

Because the shape of the glass shell is a spherical segment, *V_g_* can be described by the mathematical formula

(6) $$V_{g}=\pi h_{1}^{2}\left(\frac{3R_{g}-h_{1}}{3}\right)$$

where *h*_1_ is the height of the glass shell, *R_g_* is the radius of the spherical segment, we assume the bottom radius of the glass shell is equal to the radius of etched cavity *R*_0_, and *V_g_* can also be described as another mathematical formula

(7) $$V_{g}=\pi h_{1}\left(\frac{3R_{0}^{2}+h_{1}^{2}}{6}\right)$$

By combining (6) and (7), the mathematical equation describing the relationship between *h*_1_ and *R_g_* is

(8) $$R_{g}=\frac{R_{0}^{2}+h_{1}^{2}}{2h_{1}}$$

Thus, combining (6) and (8), the height of glass shell can be derived as

(9) $$h_{1}=\frac{{\left[\left(3V_{g}+\sqrt{{R_{0}}^{6}\pi^{2}+9{V_{g}}^{2}}\right)\pi^{2}\right]}^{2/3}-{R_{0}}^{2}\pi^{2}}{\pi {\left[\left(3V_{g}+\sqrt{{R_{0}}^{6}\pi^{2}+9{V_{g}}^{2}}\right)\pi^{2}\right]}^{1/3}}$$

where $V_{g}=\left(\frac{T_{f}}{T_{b}}-1\right)V_{E}+\frac{n_{0}RT_{f}}{P}$.

## 3. Materials and Methods

**Figure 4:**
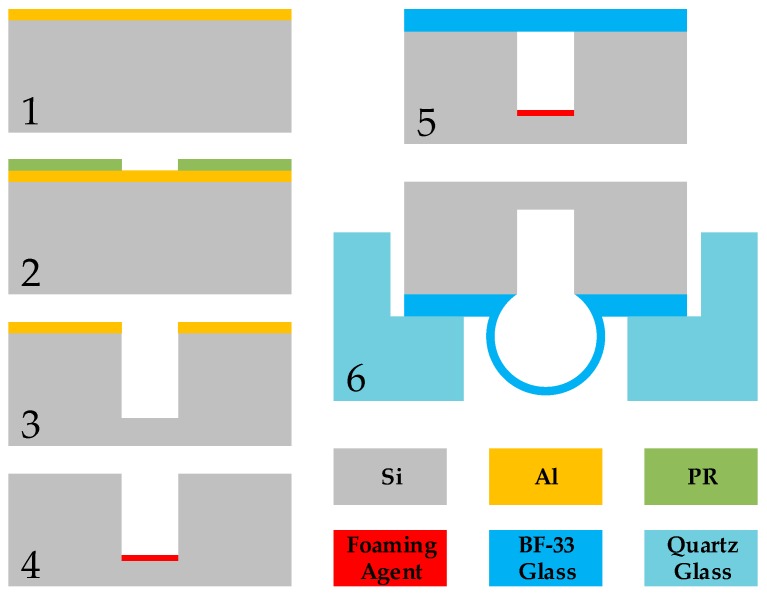
Fabrication process flow of a glass-blown spherical shell resonator.

The fabrication process flow of a glass-blown spherical shell is shown in Figure 4. First a 200-nm thick layer of aluminum is sputtered onto a 1000-μm thick silicon wafer, and then the wafer is patterned using another layer of photoresist on the sputtered layer. Next, the aluminum mask is patterned by using aluminum etching liquid and removing the photoresist by acetone, followed by etching cylindrical cavities with 300 μm in radius and 800 μm in depth by DRIE. After this step, the aluminum layer is removed by aluminum etching liquid to obtain the substrate wafer with etched cavities.

For the process of adding the foaming agent, we choose CaCO_3_ as the foaming agent because it meets the requirement of the subsequent process, with the thermal decomposition temperature (825 °C) that is lower than the temperature in the furnace (~900 °C) and higher than the temperature of anodic bonding (~400 °C). As a result, all the gas produced by CaCO_3_ can contribute to the foaming process of the glass shell during the glass blowing process. However, transferring the solid foaming agent directly is too difficult to achieve. There are two main reasons for this difficulty: one is that there is no effective method or tool to place the solid CaCO_3_ in a cavity with 300 μm radius, and the solid CaCO_3_ (especially the powders) would result in bonding surface pollution that is difficult to remove; the other reason is that CaCO_3_ is insoluble in most solvents. Thus, finding a proper means to place the foaming agent inside the micro cavity is the key problem to be solved.

Eventually, we utilized the precipitation reaction of Na_2_CO_3_ solution and CaCl_2_ solution according to the chemical equation

(10) $${\mathrm{Na}}_{2}{\mathrm{CO}}_{3}\left(\mathrm{aq}\right)+{\mathrm{CaCl}}_{2}\left(\mathrm{aq}\right)\overset{\;}{=}{\mathrm{CaCO}}_{3}\left(s\right)+2\mathrm{NaCl}\left(\mathrm{aq}\right)$$

With the reactor being the etched cavity, we use two syringe pumps (Pump 11 Elite, Harvard Apparatus, Holliston, MA, USA) and two microliter syringes (Shanghai Gaoge Industrial and Trading Company, Shanghai, China, model 1448348727, 10 μL). Moreover, we use a 34 G syringe needle (190 μm outer diameter and 60 μm inner diameter) to inject the solution because its outer diameter is smaller than the diameter of the cavity. We can control the quantity of CaCO_3_ placed in an etched cavity by adjusting the injected volume and concentration of the two reacting solutions.

After the micro-injection process, the water is removed by drying on a hot plate, thus, the quantified amount of CaCO_3_ remains in the cavity. Next, the silicon wafer is anodically bonded to a thin piece of Bf 33 glass (100 μm in thickness, Schott AG, Mainz, Germany) to isolate the atmosphere in the single cavity.

In the step of glass blowing, we invert the bonded wafer by placing the wafer upside down onto a quartz glass stencil and then transferring the assembly together into a quartz tube furnace set to a high temperature (850 °C to 950 °C); the silicon wafer and the stencil are still stable, and the foaming agent would release the gas while the glass wafer would become softened and the increased gas pressure in the cavity would force the circular thin glass film on top of the etched cavity to become a spherical glass shell. The force of gravity of the softened glass is beneficial to obtain a larger glass shell (higher H/D). After an adequate heating time (120 s to 300 s), we take the glass shell samples out of the furnace and allow the glass shell to cool down rapidly to retain its shape, because if the heating time is too short, there’s no enough time for glass to become soften and blown into hemisphere shape, and if the heating time is too long, the glass shell would deform even break because the softened glass would mostly flow to the shell vertex on the influence of gravity. The required blow-up time has been discussed in [5], we also perform series of contrast tests to determine the glass blowing characteristics.

## 4. Results and Discussion

### 4.1. Experimental Characteristics

In the experimental phase, researchers are interested in the effect of foaming agent on the shape of glass-blown shell; thus, contrast experiments were designed and executed. There are some differences between two experiments, with the most important distinction being whether the foaming agent was added into the etched cavity or not. We compare and analyze two glass shell samples we made: sample 1, which is shown in Figure 2a, was blown by a cavity without foaming agent; sample 2, which is shown in Figure 2b, was blown by a cavity that has 1.415 μg CaCO_3_ in a sealed cavity. The other parameters, e.g., the radius of cavity *R*_0_, the depth of cavity *h*, the thickness of glass *b*, the bonding temperature *T_b_*, the temperature in the furnace for glass blowing *T_f_*, and the heating time *t*, are all shown in Table 1.

The experiment results of the two samples are shown in Table 2. *V_g predicted_* is calculated by Equation (2), and *V_g real_* is calculated by Equation (6). The real volume of glass shell 2 is 276% more than the volume of shell 1, and the H/D also increases from 0.61 to 0.80, because the CaCO_3_ can release extra foaming gas during the glass blowing process. Thus, we testify that the application of chemical foaming method in fabrication of a micro-hemisphere shell resonator can help researchers achieve a larger glass shell (higher volume shell or greater height). Furthermore, we can produce a micro glass-shell with a profile closer to a sphere compared with those micro-shells blown only by the sealed atmosphere.

### 4.2. Four-Node Wineglass Resonant Frequencies Simulation

Most HRGs utilize the four-node wineglass resonant mode (*n* = 2) of the HSR to sense the change of rotation, this mode is an important and inherent characteristic that varies from dozens of kHz to several MHz for different structures, materials, and processes.

**Figure 5:**
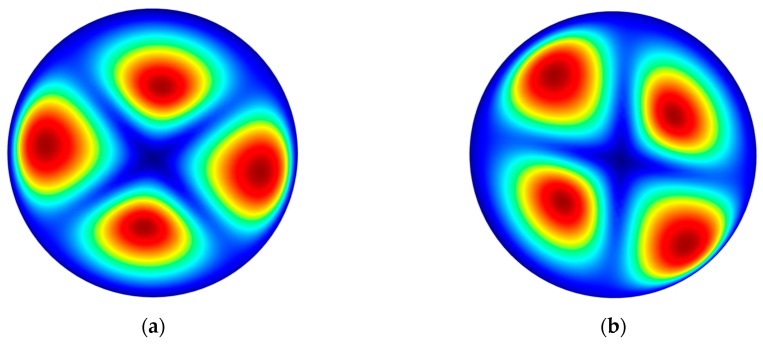
The simulated two degenerate four-node wineglass resonant mode (*n* = 2) shapes of an HSR: (**a**) The four-node wineglass resonant mode shape; (**b**) The degenerate four-node wineglass resonant mode shape.

Through the experimental process, we ultimately obtained hundreds of glass HSR samples, most of which have different H/D because of the different process parameters, and we confirmed that the most effective approach to increase the H/D is the chemical foaming method. In addition, we divided the HSR samples along the symmetry axis into two identical parts, after a detailed measurement of the HSR sectional dimensions via SEM. To study the influence of different shapes on the natural frequencies, we restructured five 3D models in SolidWorks (2010, Dassault Systèmes, Vélizy-Villacoublay, France) and then via COMSOL Multiphysics (5.2a, COMSOL Inc., Stockholm, Sweden) to simulate corresponding resonant frequencies of the four-node wineglass resonant mode. Figure 5 shows the simulated two degenerate four-node wineglass resonant mode shapes of an HSR, the phase difference between the two degenerate four-node wineglass resonant mode shapes is 45°.

The simulation results of the four-node wineglass resonant frequencies are shown in Table 3. The five samples were fabricated by using different process parameters, sample 6 and sample 7 were fabricated with the help of CaCO_3_, other parameters are same, the thickness of glass wafer *b* is 100 μm and the depth of etched cavity *h* is 800 μm. HSRs with different profiles have different four-node wineglass resonant frequencies *f_w_* (*n* = 2), we found that the *f_w_* (*n* = 2) decrease from 2.08 MHz to 0.94 MHz through the addition of CaCO_3_, it is a 54.8% decrease, enabling the HSR more sensitive to change of rotation, and it is convenient to set larger surrounding capacitive electrodes, allowing the HSR to be easier to drive and sense, showing the efficiency of the CFP method in fabrication of a micro glass HSR.

### 4.3. Glass HSR Blown by 200 μm Deep Cavity

Furthermore, we used a 500 μm silicon wafer to replace the 1000 μm silicon wafer, and we just etched 200 μm deep cavities instead of 800 μm deep cavities, the use of shallower cavities has many advantages. For example, the cost of DRIE is very high, so we can save approximately 75% of the expense of DRIE. In addition, we do not require the metal mask to resist such deep etching. Moreover, a thinner silicon wafer is beneficial for decreasing the final thickness of the HRG, which would help in the processes of DRIE, dicing, and anodic bonding, because 500 μm silicon wafers are more commonly used in micro-machining.

**Figure 6:**
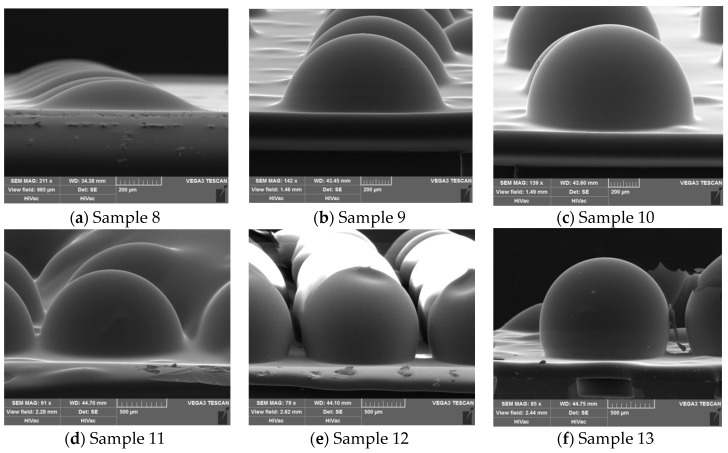
SEM images of the glass shell samples blown by 200 μm deep etched cavities.

We control the mass of CaCO_3_ in the 200 μm deep etched cavity while using the same values for the other process parameters, the SEM images of the glass shell samples are shown in Figure 6, and the corresponding results are shown in Table 4.

**Figure 7:**
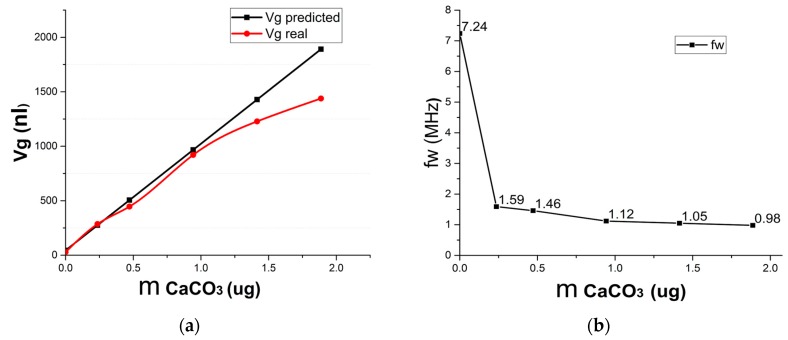
The effect of CaCO_3_ on the volume and four-node wineglass resonant frequencies of HSR samples: (**a**) Calculated volume versus real volume of HSR samples; (**b**) The four-node wineglass resonant frequencies of the HSR samples.

By comparing the calculated volume and the real volume of the HSR samples in Figure 7a, we find that the real HSR volume increases when more CaCO_3_ is added into the etched cavity. However, the increase slope decreases. For example, the real volume of shell sample 13 (which was blown by a 200 μm deep etched cavity with 1.887 μg of CaCO_3_) is 51.48 times that of sample 8 (which was formed without the CaCO_3_), although it should be 67.71 times greater in theory. The reason for this discrepancy may be the larger HSR shell has a thinner glass wall and more surface area, when we take the shell sample out of the furnace, the inner atmosphere of the HSR shell cool down more easily, which could result in the glass HSR shell shrink before the glass solidifies.

The four-node wineglass resonant frequencies of HSR samples are shown in Figure 7b. Sample 8, which was blown by an etched cavity without CaCO_3_, has a four-node wineglass resonant frequency at 7.24 MHz, and sample 9, which is blown by etched cavity with 0.236 μg CaCO_3_ that practically provides an extra 9.23 times volume as that of sample 8, has a four-node wineglass resonant frequency at 1.59 MHz, which is 78% lower than that of the control group of sample 8 (7.24 MHz). The reason for the decrease of *f_w_* (*n* = 2) is that the larger HSR has a thinner glass wall and lower stiffness. Note that the *f_w_* (*n* = 2) decrease slope of other HSR samples is lower than that of samples 8 and 9, showing the efficiency of CFP method in the fabrication of the HSR. The H/D value also increases from 0.12 to 0.50 with the addition of CaCO_3_, and the profile of sample 9 is a half sphere. Regarding samples 10, 11, and 13, their H/D values all exceed 0.5, and the largest one, sample 13, has a 4/5 sphere profile. We also attempted other approaches, such as lowering the sealed temperature or increasing the blowing temperature; the improvement of the HSR volume was minor, and those approaches are limited by the process conditions, so the CFP method is a better choice when we want to get a larger glass HSR close to a sphere.

We conclude that we could obtain a larger HSR via application of the chemical foaming method, which provides extra foaming gas. In addition, the volume of the HSR blown by the 200 μm deep etched cavity with the quantified addition of foaming agent can approach (even exceed) the HSR blown by the 800 μm deep etched cavity without foaming agent. The proposed approach not only reduces process time and cost but also presents a means to reduce the dimensions of the HRG. Furthermore, the larger HSR has several advantages, for example, greater surface area is helpful for setting larger surrounding drive and sense capacitive electrodes, which can increase the HSR detection sensitivity of rotation change.

Although the efficiency and economy of the introduction of CFP have been proven in fabrication and experiment tests of the HSR, several difficulties remain need to be solved. For example, injecting two reaction solutions must be very carefully performed; thus, we should ensure the liquid does not overflow onto the surface of the silicon wafer to avoid the failure of anodic bonding. In addition, the volumes of the two reacting solutions injected are very small (25 nL to 200 nL) and must be accurate; thus, the proposed approach has a high requirement of location accuracy and injection control precision for the platform used to add the foaming agent. Moreover, there are many factors that can influence the final shape of the glass HSR, and previous processes have absolutely essential influences on the final glass blowing result; thus, exploring a standard and successful process flow is of great importance.

## 5. Conclusions

A novel approach involving the application of the chemical foaming method in the fabrication of micro-hemispherical shell resonators was developed to increase the volume of the glass-blown shell, achieving a 51.48-fold increase compared to the traditional method without the foaming agent. The larger HSR with greater surface area is helpful for setting larger surrounding drive and sense capacitive electrodes, which can increase the HSR detection sensitivity of rotation. A height model was first presented to estimate the shape of the glass-blown shell with chemical foaming agent, and comparison experiments were performed to prove the efficiency of the new approach. We also developed a method to miniaturize the dimension of the glass HSR; this method can reduce the cost and increase the sensitivity of a glass HSR. This method may inspire related research in the design and fabrication of HRG.

## Figures and Tables

**Table 1:** The experimental parameters of the two samples.

Parameter	*R*_0_ (μm)	*h* (μm)	*b* (μm)	*T_b_* (°C)	*T_f_* (°C)	*t* (s)
Sample 1	300	800	129.82	360	900	180
Sample 2	300	800	132.85	400	900	180

**Table 2:** The experimental results of the two samples.

Parameter	*V_g predicted_* (nL)	*V_g real_* (nL)	*h*_1 *predicted*_ (μm)	*h*_1 *real*_ (μm)	*R_g real_* (μm)	H/D
Sample 1	192.92	207.69	592.85	512.12	422.78	0.61
Sample 2	1530.10	781.04	1400	950.19	592.09	0.80

**Table 3:** The simulation results of four-node wineglass resonant frequencies.

Parameter	*m*_CaCO_3__ (μg)	*R*_0_ (μm)	*T_b_* (°C)	*T_f_* (°C)	*t* (s)	*h*_1_ (μm)	*R_g_* (μm)	H/D	*f_w_* (MHz)
Sample 3	none	300	400	950	210	324.41	452.26	0.36	2.08
Sample 4	none	300	360	890	180	505.03	434.35	0.58	2.00
Sample 5	none	300	360	930	180	501.84	439.48	0.57	1.88
Sample 6	1.415	300	400	900	180	989.99	633.14	0.78	1.11
Sample 7	1.887	500	400	900	180	1039.45	759.33	0.68	0.94

**Table 4:** The experimental results of the glass shell samples blown by 200 μm deep etched cavities ^1^.

Parameter	*m*_CaCO_3__ (μg)	*V_g predicted_* (nL)	*V_g real_* (nL)	*h*_1 *predicted*_ (μm)	*h*_1 *real*_ (μm)	*D_g real_* (μm)	H/D	*f_w_* (MHz)
Sample 8	none	43.683	27.93	250.66	133.39	1088.34	0.12	7.24
Sample 9	0.236	274.73	285.81	695.78	516.26	1026.86	0.50	1.59
Sample 10	0.472	505.78	446.36	897.76	682.42	1065.13	0.64	1.46
Sample 11	0.943	966.89	920.78	1200	823.83	1412.92	0.58	1.12
Sample 12	1.415	1429	1228.41	1300	1134.62	1363.88	0.83	1.05
Sample 13	1.887	1891.1	1437.96	1500	1205.46	1433.61	0.84	0.98

^1^ These samples have same process parameters except for the mass of CaCO_3_ in the 200 μm deep etched cavity, *R*_0_ = 300 μm, *h* = 200 μm, *T_b_* = 400 °C, *T_f_* = 920 °C, *t* = 180 s.
